# Formative evaluation of the telecare fall prevention project for older veterans

**DOI:** 10.1186/1472-6963-11-119

**Published:** 2011-05-23

**Authors:** Isomi M Miake-Lye, Angel Amulis, Debra Saliba, Paul G Shekelle, Linda K Volkman, David A Ganz

**Affiliations:** 1 VA Greater Los Angeles HSR&D Center of Excellence, 16111 Plummer Street, Sepulveda, CA 91343, USA; 2 David Geffen School of Medicine at the University of California at Los Angeles, 10833 Le Conte Avenue, Los Angeles, CA 90095, USA; 3 Network 22 Telecare, Veterans Affairs Veterans Integrated Service Network 22, 11301 Wilshire Boulevard, Los Angeles, CA 90073, USA; 4 Geriatric Research, Education and Clinical Center, Veterans Affairs Greater Los Angeles Healthcare System, 11301 Wilshire Boulevard, Los Angeles, CA 90073, USA; 5 Borun Center for Gerontological Research, University of California at Los Angeles and Los Angeles Jewish Home, 10945 Le Conte Avenue, Suite 2339, Los Angeles, CA 90095, USA

## Abstract

**Background:**

Fall prevention interventions for community-dwelling older adults have been found to reduce falls in some research studies. However, wider implementation of fall prevention activities in routine care has yielded mixed results. We implemented a theory-driven program to improve care for falls at our Veterans Affairs healthcare facility. The first project arising from this program used a nurse advice telephone line to identify patients' risk factors for falls and to triage patients to appropriate services. Here we report the formative evaluation of this project.

**Methods:**

To evaluate the intervention we: 1) interviewed patient and employee stakeholders, 2) reviewed participating patients' electronic health record data and 3) abstracted information from meeting minutes. We describe the implementation process, including whether the project was implemented according to plan; identify barriers and facilitators to implementation; and assess the incremental benefit to the quality of health care for fall prevention received by patients in the project. We also estimate the cost of developing the pilot project.

**Results:**

The project underwent multiple changes over its life span, including the addition of an option to mail patients educational materials about falls. During the project's lifespan, 113 patients were considered for inclusion and 35 participated. Patient and employee interviews suggested support for the project, but revealed that transportation to medical care was a major barrier in following up on fall risks identified by nurse telephone triage. Medical record review showed that the project enhanced usual medical care with respect to home safety counseling. We discontinued the program after 18 months due to staffing limitations and competing priorities. We estimated a cost of $9194 for meeting time to develop the project.

**Conclusions:**

The project appeared feasible at its outset but could not be sustained past the first cycle of evaluation due to insufficient resources and a waning of local leadership support due to competing national priorities. Future projects will need both front-level staff commitment and prolonged high-level leadership involvement to thrive.

## Background

One quarter to one third of people aged 65 years and older experience at least one fall annually[1]. The effects of falls can impact daily life for community-dwelling older adults (those living independently outside of nursing homes or facilities that provide similar levels of care) [2,3]. Falls, or even the fear of falling, can cause older people to limit their activities, reducing their independence and self-reliance. Although research-based multifactorial fall prevention programs and exercise have demonstrated reduced falls among community-dwelling older adults [4,5], implementation of programs incorporating fall prevention activities in routine practice has produced mixed results [6-9]. A strong evidence base has not been sufficient by itself to spur uniform development or maintenance of effective fall prevention programs, and the quality of care for older people with falls and mobility disorders remains suboptimal when compared to general medical conditions such as diabetes and hypertension [10].

We developed a fall prevention program for community-dwelling older adults in the Veterans Affairs Greater Los Angeles Healthcare System (GLA) [11] building on multiple theories, including continuous quality improvement[12], diffusion of innovation theory[13], and Oliver's "Strategic Responses to Institutional Processes"[14]. A previous publication details the program development process and its theoretical basis [11]. This program serves as an umbrella for ongoing fall prevention projects at GLA. Here we report on the formative evaluation of the first project emerging from this program: use of a nurse telephone-based outreach service to assess patients' risk factors for falls and refer these patients to appropriate services. In this evaluation we describe the implementation process, including whether the implementation occurred as planned, identify the barriers and facilitators to implementation, and assess the quality of care for patients in the project.

## Methods

### Ethics Approvals

This study conforms to the ethical principles in the Helsinki Declaration, and received ethics approval from GLA (PCC 2009-010018) and the University of California at Los Angeles (G08-06-103-02) Institutional Review Boards. Because of the minimal risk nature of this project, the Institutional Review Boards waived the requirement for documentation of informed consent.

### Setting

The United States Department of Veterans Affairs (VA) is an integrated healthcare delivery system for people who are discharged from active military service [15]. Since the 1990 s the VA has had a strong tradition of quality of care measurement and improvement [16]. Quality improvement initiatives are often tied to External Peer Review Program (EPRP) data, which involves medical record review by an external contractor and is available at the level of individual healthcare facilities, allowing for peer comparisons and national and regional benchmarks [17]. VA facilities are motivated to improve their care because of financial incentives to their senior managers for meeting certain quality goals and a desire to perform well compared to other facilities. Although EPRP quality measures for falls exist, they are not in the top tier of quality measures for which results are tied to financial and non-financial incentives (e.g., prestige) to senior managers. Examples of top tier indicators applicable to older patients include use of ACE inhibitors or angiotensin receptor blockers for patients with systolic heart failure, achieving a target blood pressure of less than 140/90 mmHg in patients with hypertension, and immunization against influenza. Thus the fall prevention program we developed, although viewed in a positive light, was not a prime target for additional investment of resources.

GLA is the largest healthcare system in the VA, and is composed of ten community based outpatient clinics (CBOCs), two larger ambulatory care centers that provide both primary care and some specialty services, and a medical center that provides ambulatory, inpatient and some post-acute care. GLA also has community living centers that provide nursing home care at two of its sites. Finally, GLA either directly provides, or contracts with other organizations to provide, home care services and adult day health care. Spanning five counties in Southern California, GLA's service area includes 1.4 million veterans [18]. GLA's patients are disproportionately older than is typical in non-VA primary care settings, making falls a salient issue for our system. The system is geographically dispersed; the farthest CBOC is nearly 200 miles from the main medical center, where the investigators for this project are based. Because the CBOCs offer mostly primary care, veterans served by these CBOCs may need to travel to the medical center or an ambulatory care center for certain specialty services.

Prior to the inception of our quality improvement program, GLA had an array of fall prevention services located at its core facilities, including a fall prevention clinic, multidisciplinary geriatrics clinic, physical and occupational therapy (including a Tai Chi class), exercise programs, and home safety evaluations (through home care services). However, there was evidence that these programs were not translating into adequate quality of care for fall prevention at the population level, given GLA's suboptimal performance on the population-based quality indicator of asking outpatients age 75 and older about the presence or absence of falls in the past year [11]. Although improving case-finding for patients at risk for falls was one of GLA's priorities based on quality indicator data, developing a system to manage patients found to be at high risk for future falls was important to establish first, so that a system would be in place to handle patients identified by future case-finding efforts.

GLA is one of five healthcare systems in the VA Desert Pacific Healthcare Network, which serves Southern California and Southern Nevada. The Desert Pacific Healthcare Network both finances and directly provides some operations in each of the five systems. The Network's operations include Telecare, a nurse advice line whose primary function is to receive and triage incoming patient calls. Patients can call Telecare via a toll-free number with any concerns, including symptoms. Telecare also has a specific service, Telecare Tuck-In, which at the time of the falls project's inception was operated only for GLA. Telecare Tuck-In enables healthcare professionals to refer patients for non-urgent follow-up or check-ins with a nurse via telephone. In principle, the Tuck-In service could help mitigate some of the geographic barriers patients face in accessing services at GLA by substituting phone care for some in-person care. It is through Telecare Tuck-In that our project (described below) operated.

### Description of the project being evaluated

Here, we briefly describe how the quality improvement program began (for more details, see [11]), but subsequently focus on the formative evaluation of a specific project implemented within the umbrella of our program: the Telecare fall prevention pilot project. Although the quality improvement program and the Telecare fall prevention project were initiated simultaneously, the program ultimately became an umbrella for various fall prevention activities throughout GLA. For clarity, we use the word "program" to focus on the umbrella quality improvement initiative, and "project" to indicate the Telecare fall prevention project.

### Program development

Program development consisted of an initial leadership meeting with local GLA leaders (February 2008) to see whether fall prevention was a priority, monthly falls workgroup meetings (starting in April 2008) to decide on a specific project, and a second leadership meeting (October 2008) to review and refine the workgroup's care model for the project. Telecare Tuck-In was selected as a platform for the project during a workgroup meeting.

### Implementation

Patients participated in the project through one of two routes: 1) GLA healthcare professionals referred them to Telecare Tuck-In for fall prevention, or 2) the Tuck-In nurse was notified by colleagues of patients who had called in to Telecare with a fall or fall-related injury. The clinical champion for the project, a Tuck-In nurse, then called the patient and read scripted questions to the patient (or caregiver) to assess fall risk factors (see Additional File 1: Telecare Tuck-In nurse script for falls prevention project). The patient's answers to the script were placed into the patient's electronic health record, along with free-text notes. Based on the patient's answers to the script, the nurse determined which, if any, referrals to make for further care, using a predetermined algorithm [11]. Options included the falls clinic at the main facility, geriatrics clinic at the main facility or a separate ambulatory care center, or referral to home care services for a home safety evaluation.

### Formative evaluation

In order to obtain feedback on how to improve the project, we planned a formative evaluation, defined as an ongoing evaluation process integrated into the entire lifespan of a project, including development and implementation, rather than a classic evaluation at the end of the project's lifespan [19]. The evaluation's goals included 1) documenting how the project was implemented, including whether it was implemented and sustained according to plan, 2) identifying barriers and facilitators to implementation, and 3) studying how patients' quality of care was affected by the project. To address these aims we used three data sources: semi-structured interviews with patient and employee stakeholders, a review of workgroup meeting minutes, and electronic health record review for participating patients.

#### Semi-Structured Interviews

Semi-structured interviews were iteratively developed and refined by the research team with support from an internal GLA expert on interview design, resulting in standard interview guides (See Additional File 2: stakeholder interview script and response form and Additional File 3: patient interview script and response form). In keeping with the formative nature of the evaluation, research team members discussed interview results multiple times during the interview process, and pertinent results were shared at a monthly falls workgroup meeting on February 19, 2010; we did not, however, analyze interview data using a formal process. From our interview notes we counted up the number of times that various themes were mentioned by interviewees. Themes were not prespecified, but we made note of emergent themes during the interview process.

We identified a convenience sample of employee stakeholders for employee interviews; stakeholders were defined as those who had significant interaction with the project, including individuals who participated in the leadership meetings, were part of the workgroup, referred patients to the project, or received referrals from the project. Stakeholders were invited to participate by e-mail, and if they agreed, were interviewed by phone. The interviews, taking place between October 2009 and February 2010, covered employees' connection to the project, what they viewed the purpose of the project to be, how effective they found the project, what recommendations they might have, as well as any strengths or weaknesses they noted while working on the project. Interviews ranged from roughly two to thirty minutes in length, depending on the participants' responses and extent of involvement with the project.

Patients were interview candidates if the Tuck-In nurse had read them the script asking about falls risk factors between October 2008 and June 2009. The clinical champion maintained a log of all patients who were considered for the project and those who were actually read the script. We recruited, both by mail and by telephone, all eligible patients who had been read the script. We interviewed patients by phone in October 2009, seeking to capture their impressions of the project, with questions asking whether they remembered receiving the phone call about fall prevention, how they felt the project had helped them, and what suggestions or changes they would recommend. Patients were then asked permission for the second component of the evaluation, the electronic health record review. Patient interviews were of similar length to the employee interviews.

#### Electronic Health Record Review

For the structured electronic health record review, we adapted forms from a similar review in a previous project [20]. The review forms collect data that allow the Assessing Care of Vulnerable Elders-3 (ACOVE-3) quality indicators for falls [21] to be measured (see Additional File 4: Chart abstraction indicators). The ACOVE-3 indicators assess the extent to which the Telecare falls project complemented the quality of routine medical care for falls by specifically noting those recommended care processes that were provided by the Telecare nurse but not provided in the course of patients' usual care.

#### Cost Estimation

An additional component of the evaluation involved estimating basic costs associated with project development. Using the meeting minutes as our data source, we estimated a minimum cost of employee time for initial project development, since this activity occurred outside of employees' usual duties. The time spent by the primary investigators to evaluate this project was funded through grants. However, most of the time costs associated with project development were implicitly borne by GLA by allowing employees to participate in meetings. In order to calculate a minimum estimate of the personnel costs associated with project development, we tracked personnel attendance at the two leadership meetings and the seven workgroup meetings that occurred in the months in between these two meetings. We used actual federal employee salary data from the U.S. Office of Personnel Management [22], and thus calculated the total hourly contributions of participating workgroup and leadership meeting members. Salaries were from Fiscal Year 2008, and adjusted to include 30 percent fringe benefits standard in VA budgeting. These estimates constitute a lower bound on the overall resources used for project development, since activities of research personnel (including managing monthly workgroup meetings, developing the Telecare Tuck-In nurse script, and organizing leadership meetings) were not included. Also not included are the costs associated with actual program implementation, since we did not track times for phone calls and script administration.

## Results

### Project experience

**Figure 1:**
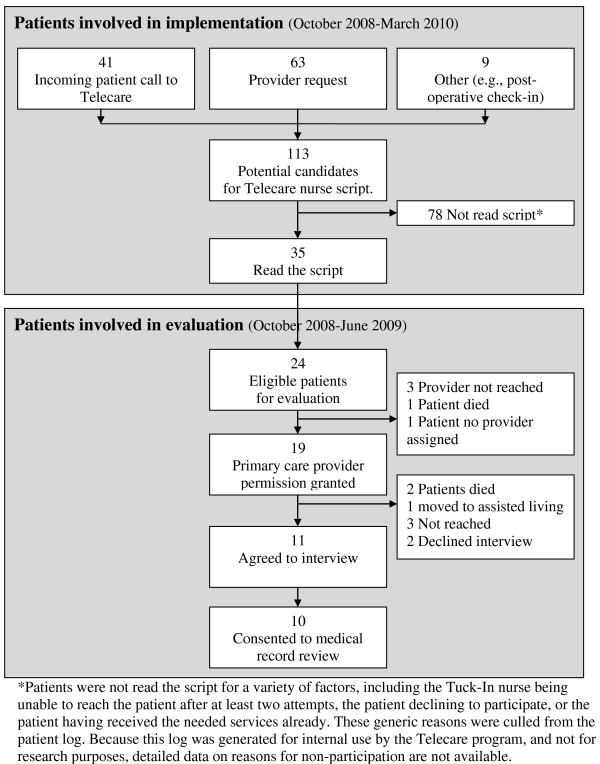
**This figure describes the flow of patients through the Telecare fall prevention program and evaluation**.

During the course of the Telecare fall prevention pilot project (October 2008 through March 2010), 113 patients were identified as potential candidates for being read the script (See Figure 1 for flow). Thirty-five patients age 65 or older were read the falls script, which was stored as a Word document that could be pasted into a progress note in the electronic health record.

The Telecare fall prevention project's end in March 2010 coincided with the retirement of its clinical champion and a dramatically increased workload for the Telecare Tuck-in service without an increase in staffing. The increased workload occurred because Telecare Tuck-In service expanded to the entire Desert Pacific Healthcare Network, and a new directive from Washington, D.C. mandated that Telecare call all patients discharged from the hospital in the network.

### Formative evaluation

A review of the meeting minutes showed that iterative changes were made throughout the implementation period (See Table 1). Major successful changes were demonstrated in the inclusion of falls-related patient education materials (See Additional File 5: Falls educational materials cover letter) [23-26], a checkbox for fall prevention in the electronic health record Telecare Tuck-In consult, and expanding the reading of the fall prevention script to post-operative patients being called by Telecare Tuck-In. However some initiatives, such as patient referrals from social workers and the Emergency Department, were developed and vetted but ultimately never fully implemented, for reasons discussed later. Throughout the project, the principal investigator met quarterly with Telecare staff to discuss any issues facing the program, and to act as liaison between the falls quality improvement workgroup and the Telecare staff. The monthly workgroup meetings are still ongoing, supporting other fall prevention quality improvement activities for GLA.

**Table 1:** Iterative Changes throughout the Program

*Date *	*Change *
	**Successfully Implemented**

December 2008	*Inclusion of falls-related patient education materials in the Telecare Tuck-In nurse's script * If the patient indicated that the materials were desired, the Patient Education Resource Center coordinator would mail out a packet of materials specifically selected by the falls workgroup developed for this project, with a cover letter (see Additional File 5)[23-26].

December 2008	*Inclusion of a check-box for fall prevention in the electronic Telecare-Tuck-In consult * Before this, healthcare professionals had to specifically request a fall prevention referral using free text in the electronic consult form.

August 2009	*Telecare Tuck-In nurses began reading the falls script to some patients who were called for post-operative check-ins * This is a separate service the Telecare Tuck-In program provides.

	**Not Fully Implemented**

October 2008 through January 2009	*Two initiatives for patient referrals were developed and vetted by the workgroup, but they were never fully implemented * • Social work service: they requested that only medical providers actually order the referrals, even if the social worker identified the patient as being at risk. • Emergency Department: although one of us briefed the Emergency Department physicians on the program and one or two test referrals were made, we had difficulty sustaining interest in the program.

For the patient interviews, among 24 eligible patients who participated in the project between October 2008 and June 2009, 11 patients were interviewed, of whom 10 consented to electronic health record review (See Figure 1 for flow). Of these 10, nine participants were male, six were from community based outpatient clinics (CBOCs), and nine were living in private residences. We discuss medical record data from nine participants who experienced a fall below. Of the 12 employees contacted for interviews, two were unable to participate due to scheduling difficulties; thus, 10 employees participated in interviews.

#### Interview Data: Facilitators

In their interviews, three employees mentioned good communication as a strength of the project. Four employees perceived the project to be based on a strong model, which was easy to use and had the potential to be spread to other locations. Four employees also noted that the project enabled a wider reach to patients, including patients who might not otherwise be available for in-depth follow-up. Six employees stated that it might be too early to make definitive statements about the project's impact. Five patients noted the following project advantages: making them well informed about falls, helping them feel prepared with home safety and prevention advice, and causing them to be more alert to the issue of falls. Two patients also mentioned the emotional benefit of the project, which made them feel cared about.

#### Interview Data: Barriers and Suggestions for Improvement

These key strengths also related to the main barrier facing this project: patient transportation. Because we were able to reach patients by telephone for whom transportation to an appropriate clinic was not feasible, our project included those who would otherwise be excluded from many services. Although this project feature in and of itself was emphasized as a success by patients and employees alike, patients who were found to need in-person follow-up services then faced a dilemma. A total of five interviewees (both employees and patients) cited the dilemma of remote locations. The other main barrier, noted by six people (patients and employees combined) related to limited resources, specifically the tight schedules and large caseloads of both Telecare and the VA system in general. Finally, two employee stakeholders also agreed that the project needed better recognition, but with healthcare providers.

Employee stakeholders offered creative solutions to the barriers above. For the transportation issue, suggestions included the creation of a DVD of fall prevention information for CBOC staff and eligible patients, and more ancillary staff at CBOCs and other locations who could provide more local support for the falls project. Suggestions for the resource limitations included adding more resources and support, mentioned by five employees. For improving project recognition, employees suggested more dissemination of program information and the inclusion of a variety of providers within GLA.

#### Interview Data: Other Findings

Although they were happy with their care, two patients were not able to immediately distinguish between this project and other VA services, specifically phone calls, they received. In fact, three others cited duplication of services, saying that they received calls that covered similar material to what they had discussed before, either with their physicians or other healthcare professionals both within and outside the VA. A total of eight patients had trouble remembering the call, even though in all cases a call was documented in their records. This may relate to issues with recall (in some cases interviews occurred a number of months after the index phone call to the patient where the fall prevention script was read).

Our stakeholder interviews brought to light several possible contributing factors to the project's wind down. We found that first, Telecare nurses perceived that a low number of patients followed through with recommended care, due to a lack of transportation or other local care options (e.g., physical therapy or geriatric medicine). Second, when the project's clinical champion planned to transition out of the VA, it was implicitly decided to wind down the project, rather than train a replacement, given the large workload of the Telecare staff. However, the Telecare manager did mention a second life for the project. Once Telecare stabilized with its new workload, she thought that the project could be revisited on the expanded network-wide scale.

#### Electronic Health Record Review

Usual health care completely fulfilled two of the quality indicators for all eligible patients: documenting a basic falls history (nine out of nine eligible patients) and evaluation for/prescription of a new assistive device (one out of one eligible patients, see Table 2). Telecare effectively supplemented gaps in usual health care on three other indicators: vision check (usually through documentation of care provided outside the VA), home safety, and review of an existing assistive device. However, for the two indicators that require face-to-face interaction-checking orthostatic vital signs and gait, balance and strength examination - Telecare was not expected to complete these indicators over the phone, and neither of these indicators was fulfilled for a number of eligible patients through usual health care. Although gaps were left by both usual health care and Telecare on the indicators relating to cognition and exercise programs/physical therapy, all indicators were fulfilled a majority of the time, except for orthostatic vital signs.

**Table 2:** Chart Abstraction Data

Indicator measured	N Eligible	Completed by usual care	VA Telecare Number completed/number eligible*
Fall history	9	9	9/9

Orthostatic vital signs	9	3	3**/9

Vision	9	4	8/9

Gait, balance, strength	9	5	5**/9

Cognitive evaluation	9	4	6/9

Home safety	9	4	9/9

New assistive device	1	1	1/1

Review assistive device	7	6	7/7

Exercise program or physical therapy	5	3	3/5

* "Number completed" refers to the number of patients for whom a given care process was completed. "Number eligible" refers to the number of patients who were eligible for a given care process.

** Because Telecare is a phone-based program, these measures were not applicable.

#### Project development cost estimation

We estimated the basic cost of employee time for project development to be $7072 for salary alone and $9194 when counting salary plus fringe benefits. These numbers do not include implementation costs.

## Discussion

### Project strengths

Flexible implementation and an iterative approach to change were two important strengths of the project. For example, in opting to keep the Telecare nurse script as a Word document outside the electronic health record, we were able to make updates and changes more easily (Table 1) than if the script were embedded in the electronic health record. However, this may have resulted in less institutionalization of the script in Telecare's routine. An additional strength related to the telephonic mode of outreach: because patients were typically at home when called, and potentially just after their fall, they were able to provide information that might have been forgotten, or not discussed, at a medical office visit. This strength was particularly noticeable in Telecare's home safety counseling.

### General lessons learned

Leadership support is vital to sustaining any project [12]. Although we had support within GLA for our project, network or national leadership support might be needed for a successful reintroduction of the project. In addition, having support from a clinical champion and the Telecare manager was critical in implementation, and would need to be present in the next iteration of the project. Overall, a more supportive context would be necessary to restart and maintain the project [27].

### Lessons learned specific to telephone-based projects

Previous work has shown mixed results for telephone-based consultation and triage services, with scant information about implementation issues [28]. Some studies showed that telephone consultation services resulted in decreased workload for primary care providers, while others found the opposite; the same mixed picture exists for the effect of telephone consultation on costs. Our experience implementing the Telecare fall prevention pilot project suggests that geographic factors are very important in determining the effectiveness of telephone consultation and triage. Although we were able to reach patients who were far from specialized VA services, we also had difficulty in triaging them to needed resources once we identified their care needs, precisely because of their distance from care. Similar geographic constraints affect other VA facilities as well [29]. Screening patients for geographical proximity to care could have mitigated some of the frustration associated with transportation. By taking their location into account we could have made more realistic referrals.

For subsequent work we also need to promote increased awareness of our project beyond what was done during the pilot. Although we involved social work service and the Emergency Department, we did not have the resources (in terms of time and people) to develop these connections to the degree necessary to follow through, build relationships, and institutionalize the project.

We also need to reconsider our target population and mode of outreach. By using Telecare, we were able to reach more patients than could be reached with face-to-face office visits, but this population still does not include those who have no interaction with the healthcare system, since patients at a minimum had to either call Telecare or see a GLA provider to be included in the project. Similar conclusions have been reached by others engaged in telephone outreach programs [30]. Community linkages to non-healthcare venues, such as senior centers, could be an important complement to our current medical care focus.

## Conclusion

As the Telecare fall prevention project wound down, the formative evaluation took on a new meaning. Rather than using the feedback for further iterative change, the evaluation also served as an exploration of factors that contributed to the discontinuation and lack of sustainability. We intended the project to function in a continuous quality improvement model, but were not able to support the project long enough to implement changes in response to the first full cycle of evaluation. The *why *and *how *of quality improvement implementation is just as important to document as whether the project succeeded [12]. We briefly speculate on lessons learned below.

First, this project's low profile and limited resource base made it difficult to compete with "top-down" (that is, dictated from higher administrative levels) projects that were a higher priority for the facility, such as improving influenza vaccination rates. The "bottom-up" (locally initiated) approach has been suggested as having benefits like more staff buy-in, intrinsic reward for local staff, and a more customized and detail-oriented implementation [31], but when placing a bottom-up project in direct competition with a top-down project, these factors may not be sufficient. We did seek leadership support at the outset during project development, but mostly functioned using a bottom-up approach thereafter. Using a top-down approach involving network and even national leadership may have shielded our project from the consequences of Telecare's shift in priorities. With growing attention at a national level, fall prevention is gaining support from coordination within the Office of Geriatrics and Extended Care and new falls quality indicators are being piloted. This top-level support may improve chances of success for future efforts of the fall prevention program. Hybrid top-down/bottom-up models are advocated as the most effective way to implement quality improvement projects, but given the quickly evolving field, there are no reliable guidelines for applying quality improvement methods to any specific context [12,31].

Beyond top-level support, more resources would be another key factor in future iterations of this project. Our low response rate reflects the realities of the multiple competing demands of routine clinical care. Because there were no resources specifically allocated for this project, the Telecare staff involved did not have the capacity to be overly inclusive with respect to screening patients. Our impression is that an internal triage occurred to target the patients at highest need given the time constraints - patients already receiving appropriate services were lower priority and may not have been called.

An unintended positive byproduct of this project was the development and continuation of monthly workgroup meetings to discuss quality improvement activities around fall prevention more generally. Although this specific project had a limited life-span, new projects are ongoing, which we hope will further the original mission to improve the quality of care for fall prevention at our facility

## Competing interests

The authors declare that they have no competing interests.

## Authors' contributions

IML, DS, PGS and DAG designed the formative evaluation. AA and LKV oversaw the operations of the Telecare fall prevention pilot program, and AA implemented the pilot program. IML conducted interviews, and DAG reviewed electronic health record data. IML and DAG analyzed meeting minutes and drafted the manuscript. All authors provided comments for critical revision of the manuscript, and read and approved the final manuscript.

## Funding

This work was funded by the U.S. Department of Veterans Affairs, Veterans Health Administration, VA Health Services Research & Development (HSR&D) Service through the VA Greater Los Angeles HSR&D Center of Excellence (Project # VA CD2 08-012-1); and the John A. Hartford Foundation/UCLA Geriatrics Center of Excellence.

The views expressed in this article are those of the authors and do not necessarily reflect the position or policy of the U.S. Department of Veterans Affairs.

## Pre-publication history

The pre-publication history for this paper can be accessed here:

http://www.biomedcentral.com/1472-6963/11/119/prepub

## Supplementary Material

Additional file 1 **Telecare Tuck-In nurse script for falls prevention project**. The script used by Telecare Tuck-In nurses during the falls prevention project. Click here for file

Additional file 2 **Stakeholder interview script and response form**. The script and response form used during the stakeholder interview data collection. Click here for file

Additional file 3 **Patient interview script and response form**. The script and response form used during the patient interview data collection. Click here for file

Additional file 4 **Chart abstraction indicators**. A description of the indicators collected during chart abstraction. Click here for file

Additional file 5 **Falls educational materials cover letter**. The cover letter sent to patients with educational materials during the falls prevention project. Click here for file
