# Supplementary material for: Formative evaluation of the telecare fall prevention project for older veterans
Source: BMC Health Serv Res. 2011 May 23;11:119. doi: 10.1186/1472-6963-11-119 (PMC3127979; doi:10.1186/1472-6963-11-119)
Supplement: Additional file 1 — Telecare Tuck-In nurse script for falls prevention project. The script used by Telecare Tuck-In nurses during the falls prevention project. [file 1472-6963-11-119-S1.DOCX]

Additional File 1. Telecare Tuck-In nurse script for falls prevention project

**1a. In the past year, have you fallen? Yes** **No**

**1b.** (If yes to 1a) **With the fall, did you hurt yourself or need to see a doctor because of a fall? Yes No**

**2a.  Do you have problems with walking or balance? Yes No**

**2b.** (If yes to 2a) **Would you like a medical expert to check your walking and balance to see if you can improve them? Yes No**

**3a.  Do you use a walking aid, such as a cane or walker?** **Yes No**

**3b.** (If yes to 3a) **Has a doctor, nurse or therapist showed you how to use your cane or walker?** **Yes No**

**3c.** (If no to 3a) **Has a doctor, nurse or therapist recommended you use a cane or walker?** **Yes No**

4.  (Review medication list in CPRS. Assess for use of benzodiazepines, such as temazepam, lorazepam, diazepam. If a patient is listed as taking a benzodiazepine, ask the following:)

**4a. Are you still taking a medicine called [name of medicine]? Yes No**

**4b.** (If yes to 4a) **Would you be willing to meet with a doctor to talk about whether you still need to be on this medicine? Yes No**

**5a.  Do you feel dizzy, woozy, or lightheaded when you sit up or stand up? Yes No**

**5b.** (If yes to 5a) **Has this happened more than 4 times in the past month?**

**Yes No**

**5c.** (If yes to 5b) **Would you be willing to see a doctor for this problem?**

**Yes No**

6.  (Look up whether the patient has had an eye exam within the past year in CPRS. If not, ask the following:)

**6a. Do you have problems with your vision? Yes No**

**6b.** (If yes to 6a) **When was your last appointment with an eye doctor?**

**Record date here:** **Today’s date:**

(If no appointment at the VA or an outside provider in the past year, ask the following:)

**6c. Would you like to see an eye doctor to check your vision? Yes No**

**7a.** **Do you need help to use the bathtub, shower, or toilet?** **Yes No**

**7b.** (If yes to 7a) **Would you like someone to teach you ways to make using the bathtub, shower, or toilet easier? Yes No**

**7c.** (If yes to 7a) **Do you have grab bars in your bathroom? Yes No**

**7d.** (If no to 7c) **Would you be interested in having someone visit your home to see whether grab bars should be put in? Yes No**

**8a.** **Have you been having memory problems? Yes No**

**8b.** (If yes to 8a) **Would you like to see a doctor to check your memory?**

**Yes No**

**9. Are there any other problems that you’re having that you’d like to discuss with me?**

**10. Would you like more information mailed to you about how to prevent falls? Yes No**

***Clinical* Referral pathways:**

Patients are referred either to PM&R falls clinic or GRECC clinic, but not both.

A PM&R falls clinic consult should be placed if the patient has transportation to come to West LA VA and ALL of the following are true:

- “YES” answer to question 2b
- “YES” answer to either question 7b or 7d
- “NO” answer to question 8a

If the patient is not a candidate for PM&R clinic, a geriatrics consult, stating falls as the reason for consult, should be placed to either West LA GRECC clinic or Sepulveda geriatric medicine clinic, if the patient has transportation to come to West LA VA and/or Sepulveda and ANY of the following are true:

- “YES” answer to question 2b
- “YES” answer to question 4b
- “YES” answer to question 5c
- “YES” answer to question 6c
- “YES” answer to question 7b or 7d
- “YES” answer to question 8b

To expedite the consult, please call <<NAME>>, LVN (West LA <<PHONE>>) or <<NAME>>, PA (Sepulveda, <<PHONE>>). They are the clinic coordinators for the West LA GRECC and Sepulveda geriatrics clinics, respectively.

A consult for Home Care Services should be placed if the patient is unable to leave his/her home to get to the doctor’s office and ANY of the following are true:

- A “yes” answer to question 2b 🡪 order home physical and occupational therapy for gait and balance assessment and training
- A “no” answer to question 3b 🡪 order home physical and occupational therapy to review how to use cane or walker
- A “yes” answer to question 3c 🡪 order home physical and occupational therapy to evaluate need for cane or walker
- A “yes” answer to question 4b 🡪 order visiting nurse to do medication review
- A “yes” answer to question 5c 🡪 order visiting nurse to do orthostatic vital signs
- A “yes” answer to question 7b 🡪 order home occupational therapy to teach patients how to bathe safely
- A “yes” answer to question 7d 🡪 order home safety evaluation

IF THE PATIENT MEETS NONE OF THE ABOVE CRITERIA, THE PATIENT SHOULD BE REFERRED BACK TO HIS/HER PRIMARY CARE PROVIDER FOR ADDITIONAL EVALUATION.

***Health education* referral pathway:**

If a patient answers “yes” to question 10, please e-mail the relevant contact below from the Patient Education Resource Center (PERC) requesting that a fall prevention education packet be mailed to the veteran. Please include the veteran’s name and mailing address in your request:

| **PCP Location** | **PERC contact** | **E-mail** | **phone** |
| --- | --- | --- | --- |
| West LA | <<NAME>> | <<EMAIL>> | <<PHONE>> |
| Sepulveda | <<NAME>> | <<EMAIL>> | <<PHONE>> |
| LAACC or CBOC | <<NAME>> | <<EMAIL>> | <<PHONE>> |
