# Supplementary material for: Formative evaluation of the telecare fall prevention project for older veterans
Source: BMC Health Serv Res. 2011 May 23;11:119. doi: 10.1186/1472-6963-11-119 (PMC3127979; doi:10.1186/1472-6963-11-119)
Supplement: Additional file 2 — Stakeholder interview script and response form. The script and response form used during the stakeholder interview data collection. [file 1472-6963-11-119-S2.DOCX]

Additional File 2. Stakeholder interview script and response form

Hello, my name is __________. We’re calling today to ask you to take part in a research interview to improve VA’s Telecare fall prevention program. This program exists to help prevent Veterans from falling and to maintain their independence and quality of life. We want to see how this program can better meet your needs. The interview questions mostly ask about your satisfaction with the program and how it could be improved. If you can’t complete the interview now for any reason, we ask you to allow us to call you at a later date of your choosing. You can decide not to answer any particular question for any reason. *(PAUSE)*

If you agree to participate, we will take about 10-25 minutes of your time, depending on your answers. We will keep your answers confidential, which means that nothing you say will be revealed to other stakeholders besides the research team doing the study. If you agree to participate, I will be taking notes about your answers to questions but we will not be taping this conversation.

The biggest risk of participating is that it might be uncomfortable answering some of the questions. There aren’t any personal benefits from participating. We hope that your answers will help the VA improve care for older veterans. Even if you agree to participate now, you can change your mind about any of these things at any point later during the interview or after it. Either way, your decision won’t affect your rights and benefits as a stakeholder and your answers will remain completely confidential.

You may withdraw your consent at any time and discontinue participation without penalty or loss of benefits to which you were otherwise entitled.

You can choose whether or not you want to be in this study. If you volunteer to be in this study, you may leave the study at any time without consequences of any kind. You are not waiving any of your legal rights if you choose to be in this research study. You may refuse to answer any questions that you do not want to answer and still remain in the study.

Do you have any questions about the study or your participation?

- ***IF YES:*** *RESPOND TO ALL QUESTIONS BEFORE PROCEEDING*

*Stakeholder agrees to interview*

*Stakeholder declines to be interviewed*

*Stakeholder asks for interview to be rescheduled*

*(Interview starts here.)*

1) What is your connection to the Telecare fall prevention program?”

2) In your view, what is the purpose of this program?

3) In your view, is the program accomplishing its intended purpose? (*If appropriate:* If not, why not?)

4) Describe some of the barriers you face in your work with regards to this program.

5) Describe things that make it easier for you to carry out your work with this program.

6) What are your recommendations to improve the program?

7) Is there anything else you’d like to tell us?

8) Thanks very much for participating! We are grateful for your time.
