# Supplementary material for: Formative evaluation of the telecare fall prevention project for older veterans
Source: BMC Health Serv Res. 2011 May 23;11:119. doi: 10.1186/1472-6963-11-119 (PMC3127979; doi:10.1186/1472-6963-11-119)
Supplement: Additional file 3 — Patient interview script and response form. The script and response form used during the patient interview data collection. [file 1472-6963-11-119-S3.DOCX]

Additional File 3. Patient Interview Script and Response Form

Hello, my name is __________. We’re calling today to ask you to take part in a research study to improve VA’s Telecare fall prevention program.  Let me take a few more moments to explain what we will be asking you to do, so you can voluntarily decide whether or not to participate. The Telecare fall prevention program at the VA is about improving the care you receive to help prevent falls and improve balance, strength, and walking in older veterans. We want to see how this program can better meet your needs.

Deciding to participate means you agree to TWO things:

- 1. First, we ask you to complete an interview now about the Telecare fall prevention program.  Depending on your answers, this part could take between 10 and 25 minutes to complete.  The questions mostly ask your satisfaction with the program and how it could be improved. If you can’t complete the interview now for any reason, we ask you to allow us to call you at a later date of your choosing. You can decide not to answer any particular question for any reason. *(PAUSE)*
  2. Secondly, when you are done with the interview, we’re going to ask you for permission to review your medical records related to this visit. We’ll also talk to you about whether you’re willing to grant permission to be contacted about future research related to this project. You will have the opportunity to refuse these requests at that time. *(PAUSE)*

The biggest risk of participating is that it might make you tired, or you might be embarrassed or upset by some of the questions. There aren’t any personal benefits from participating, and the study doesn’t provide any medical treatment. We do hope that your answers will help the VA improve care for older veterans. Even if you agree now, you can change your mind about any of these things at any point later during the interview or after it. Either way, your decision won’t affect your rights and benefits as a VA patient and your answers will remain completely confidential.

You may withdraw your consent at any time and discontinue participation without penalty or loss of benefits to which you were otherwise entitled.

You can choose whether or not you want to be in this study. If you volunteer to be in this study, you may leave the study at any time without consequences of any kind. You are not waiving any of your legal rights if you choose to be in this research study. You may refuse to answer any questions that you do not want to answer and still remain in the study.

Do you have any questions about the study or your participation?

- ***IF YES:*** *RESPOND TO ALL QUESTIONS BEFORE PROCEEDING*

*Veteran agrees to interview*

*Veteran declines to be interviewed*

*Veteran unsure about interviewing; requests to be contacted at later date*

*Unable to reach Veteran after 3 tries*

*(Interview script starts here)*

1. Do you remember being contacted by the Telecare fall prevention program?

*(If yes, continue to question 3. If no, continue to question 2.)*

*Yes*

*No*

2. In our program, a nurse talks to you over the phone to find out if you’ve had a fall or if you might have balance or walking challenges. Do you remember having been called by the nurse?

*(If yes, continue to question 3. If no, continue to medical record consent)*

*Yes*

*No*

3. When the nurse called you, she asked you if you had fallen in the past year. How did you feel about being asked this question? (*If appropriate:* How would you have liked to be asked about falls?)

4. What is your opinion of the Telecare fall prevention program? (*If appropriate:* Why/how did you form this opinion?)

5. Has this program helped you? (*If appropriate:* In what way?)

6. Is there anything about this program you’d like to change? If so, what?

7. What stands out to you the most about this program?

8. What do you feel is the best way for the Telecare fall prevention program to reach out to you in the future?

9. Is there anything else you’d like to tell us?

*(Interviewer now consents for medical record review.)*

At this point we’d like to discuss with you whether we could review your medical records. The purpose of reviewing your records is to improve the care you’re receiving for fall prevention, as well as the care that other veterans like you receive. If you give us permission to review your records, we will only be collecting information related to fall prevention. We will keep your information confidential. Even if you agree now, you can change your mind at any time. *(PAUSE)*

(*If veteran requests more information about the medical record review process:*) Here’s some more information about how we will review your records if you give us permission. Our lead investigator, David Ganz, MD, will be collecting information from your electronic medical chart. He will be looking at the kinds of care that VA doctors and other professionals provide to you to prevent falls, and how long you had to wait to get the care you needed. He will be careful to keep your information confidential. *(PAUSE)*

Do you have any questions about the study or your participation?

*(ANSWER ALL QUESTIONS BEFORE PROCEEDING)*

*Veteran agrees to medical record review*

*Veteran declines medical record review*

*Veteran unsure; requests to be recontacted at later date*

*(Discussion about contacting Veteran for future research)*

We’d like permission to contact you for future research projects if you’re interested. Your answer will not affect your regular care at the VA in any way.

*Veteran gives permission for future contact*

*Veteran declines future contact*

*(If permission to contact for future research granted)*

What is the best telephone number to reach you at: _______________

What is the best address to reach you at:

Thank you for participating in today’s phone call. We are grateful for your time.
