# Supplementary material for: Formative evaluation of the telecare fall prevention project for older veterans
Source: BMC Health Serv Res. 2011 May 23;11:119. doi: 10.1186/1472-6963-11-119 (PMC3127979; doi:10.1186/1472-6963-11-119)
Supplement: Additional file 4 — Chart abstraction indicators. A description of the indicators collected during chart abstraction. [file 1472-6963-11-119-S4.DOCX]

Additional File 4. Chart Abstraction Indicators

*Detection of falls*

ALL vulnerable elders should have documentation that they have been asked annually about the occurrence of recent falls BECAUSE falls are common, often preventable, frequently unreported, and often cause injury and unnecessary restriction of activity, which results in a reduction in overall health and quality of life. Additionally, a recent history of falls is a potent predictor of future falls.

*Fall history*

IF a vulnerable elder reports a history of >2 falls (or 1 fall with injury) in the past year, THEN there should be documentation of a basic fall history (circumstances, medications, chronic conditions, mobility, alcohol intake) within 3 months of the report (or within 4 weeks of the report, if the most recent fall occurred in the past 4 weeks).

*Orthostatic vital signs*

IF a vulnerable elder reports a history of >2 falls (or 1 fall with injury) in the past year, THEN there should be documentation of orthostatic vital signs within 3 months of the report (or within 4 weeks of the report, if the most recent fall occurred in the past 4 weeks).

*Vision evaluation*

IF a vulnerable elder reports a history of >2 falls (or 1 fall with injury) in the past year, THEN there should be documentation of receipt of an eye exam in the past year, or evidence of visual acuity testing within 3 months of the report.*

*Gait, balance, and strength evaluation after a fall*

IF a vulnerable elder reports a history of >2 falls (or 1 fall with injury) in the past year, THEN there should be documentation of a basic gait, balance, and strength evaluation within 3 months of the report (or within 4 weeks of the report, if the most recent fall occurred in the past 4 weeks).

*Gait and balance evaluation for worsening mobility problem**

IF a vulnerable elder has new or worsening difficulty with ambulation, balance, or mobility, THEN there should be documentation of a basic gait, balance, and strength evaluation within 3 months of the report.

*Cognitive evaluation*

IF a vulnerable elder reports a history of >2 falls (or 1 fall with injury) in the past year, THEN there should be documentation of an assessment of cognitive status in the past 6 months or within 3 months of the report (or within 4 weeks of the report, if the most recent fall occurred in the past 4 weeks).

*Home safety evaluation*

IF a vulnerable elder reports a history of >2 falls (or 1 fall with injury) in the past year, THEN there should be documentation of an assessment and modification of home hazards recommended in the past year or within 3 months of the report.

*Benzodiazepine discontinuation**

IF a vulnerable elder reports a history of > 2 falls (or 1 fall with injury) in the past year and is taking a benzodiazepine, THEN there should be documentation of a discussion of related risks and assistance offered to reduce/discontinue benzodiazepine use.

*New assistive device for balance disorder*

IF a vulnerable elder demonstrates decreased balance/proprioception or increased postural sway AND does not have an assistive device, THEN an evaluation/prescription for an assistive device should be offered within 3 months.

*Review assistive device*

IF a vulnerable elder reports a history of >2 falls (or 1 fall with injury) in the past year AND has an assistive device, THEN there should be documentation of an assistive device review in the past 6 months or within 3 months of the report (or within 4 weeks of the report, if the most recent fall occurred in the past 4 weeks).

*Exercise program or physical therapy*

IF a vulnerable elder is found to have a problem with gait, balance, strength, or endurance, THEN there should be documentation of a structured/supervised exercise program offered in the past 6 months or within 3 months of the report.

* We did not report on these indicators because nobody was eligible for them in our sample.

Source of indicators: Chang, J.T. and D.A. Ganz, *Quality indicators for falls and mobility problems in vulnerable elders.* J Am Geriatr Soc, 2007. **55 Suppl 2**: p. S327-34.
