# Supplementary material for: Formative evaluation of the telecare fall prevention project for older veterans
Source: BMC Health Serv Res. 2011 May 23;11:119. doi: 10.1186/1472-6963-11-119 (PMC3127979; doi:10.1186/1472-6963-11-119)
Supplement: Additional file 5 — Falls educational materials cover letter. The cover letter sent to patients with educational materials during the falls prevention project. [file 1472-6963-11-119-S5.DOCX]

Additional File 5. Falls Educational Materials Cover Letter

(Date)

Dear (name of patient or caregiver),

Falls are a common cause of injury and disability among older adults. The enclosed materials offer several tips and strategies to prevent falls. Handouts are included on some of the following topics: exercise tips for balance and strength, making changes in your living space and moving safely using a cane or walking device. Please read these handouts with your family members, friends and caregivers. Be sure to also discuss this information with your health care providers such as your physician, nurse or physical therapist. If you have further questions about fall prevention or other health concerns, please call our 24 hour TeleCare Nurse Advice Line at X XXX XXX XXXX. For other health information or advice, please contact one of the Greater Los Angeles Patient Education Resource Centers (PERC):

- West Los Angeles PERC

XXX.XXX.XXXX ext XXXXX

Bldg. XXX, Room XXXX

- Downtown PERC

XXX.XXX.XXXX ext XXXX

Room XXXX

- Sepulveda PERC

XXX.XXX.XXXX

Bldg. XXX, Room XXXX

Sincerely,

(Name, title)
